# 

**Published:** 1887-09-03

**Authors:** 


PRICE ONE PENNY.

No. 49, Vol. II.

September 3rd, 1887.

[Registered at the General Post Office as a Newspaper.]

Per Annum, 6s. 6d., post free.

Monthly Parts, 6d.; post free, 7½d.

Ditto per Ann., 7s. 6d. post free.

